# Improved Entropy-Based Condition Monitoring for Pressure Pipeline Through Acoustic Denoising

**DOI:** 10.3390/e27010010

**Published:** 2024-12-27

**Authors:** Yu Wan, Shaochen Lin, Chuanling Jin, Yan Gao, Yang Yang

**Affiliations:** Jiangsu Frontier Electric Technology Co., Ltd., Nanjing 211102, China

**Keywords:** condition monitoring, signal entropy, pressure pipeline, acoustic denoising, abnormal sound detection

## Abstract

During long-term operation in complex environments, the pressure pipeline systems are prone to damage and faults, and serious safety accidents may occur without real-time condition monitoring. Moreover, in traditional non-contact monitoring approaches, acoustic signals are widely employed for condition monitoring for pressure pipelines, which are easily contaminated by background noise and provide unsatisfactory accuracy. As a tool for quantifying uncertainty and complexity, signal entropy is applied to detect abnormal conditions. Based on the characteristics of entropy and acoustic signals, an improved entropy-based condition monitoring method is proposed for pressure pipelines through acoustic denoising. Specifically, this improved entropy-based noise reduction model is proposed to reduce the noise of monitoring acoustic signals through adversarial training. Based on the denoising of acoustic signals, an abnormal sound detection method is proposed to realize condition monitoring for pressure pipelines. In addition, the experimental platform is built to test the effectiveness and reliability of the proposed method. The results indicate that the quality of signal denoising can reach over 3 dB, while the accuracy of condition monitoring is about 92% for different conditions. Finally, the superiority of the proposed method is verified by comparing it with other methods.

## 1. Introduction

Pressure pipelines are widely used in the industrial water and gas supply fields under harsh environments, such as high temperature, high pressure, and strong impact [1]. These conditions can easily lead to fatigue and damage to pressure pipeline, resulting in abnormal vibration and sound without timely condition monitoring. Due to the complex working environment and the background noise emitted by different devices such as fans, cooling towers, valves and so on, the quality of monitoring acoustic signals is unsatisfactory, with poor information entropy. Moreover, abnormal sound is difficult to detect and the accuracy of condition monitoring is unsatisfactory based on traditional acoustic sensors [2]. Therefore, there is an urgent need to establish systems to effectively monitor the operating conditions of pressure pipelines through abnormal sound detection, which is crucial for the safe and stable operation of pressure pipelines and transmission systems based on condition monitoring.

Currently, pipeline system condition monitoring methods mainly focus on faults such as blockages and leakages, and there is a lack of research on the detection of abnormal vibrations and sounds [3]. Generally, the pressure pipelines operate in high-temperature environments, and are wrapped with thick protective materials on the surface of the pipeline [4]. This limits the direct application of a contact monitoring sensor, such as a strain gauge and vibration sensor [5]. Conversely, acoustic sensors, as non-contact and non-destructive testing tools, are widely employed to monitor the pressure pipelines based on high-quality acoustic signals. However, the monitoring signals obtained from acoustic sensors are contaminated by background noise, which leads to the unsatisfactory application of acoustic sensor-based condition monitoring methods [6]. On the other hand, the occurrence of abnormal sound in pressure pipelines has strong randomness and suddenness with a short duration, resulting in insufficient abnormal sound data. This further limits the application of condition monitoring and abnormal sound detection in pressure pipelines [7]. Therefore, it is necessary to study noise reduction methods for effective abnormal sound detection in noisy signals, which is significant for the condition monitoring of pressure pipelines.

At present, noise reduction methods are applied through two means, including signal processing, and different denoising models [8]. Signal processing denoising methods consist of several signal decomposition and reconstruction algorithms and their improvement methods, such as empirical mode decomposition (EMD) [9], variational mode decomposition (VMD) [10], wavelet packet decomposition (WPD) [11], etc. They decompose the noisy signal by manually selecting the parameter and reconstruction method based on expertise; however, they lead to insufficient generalization and poor mobility of noise reduction. In addition, the denoising methods of low-rank matrix decomposition and expectation maximization have been proposed in recent decades and have been applied to condition monitoring, decomposing a matrix into multiple low rank matrices and evaluating maximum expectations to eliminate noise [12]. On the other hand, with the development of artificial intelligence and deep learning models, several denoising models have emerged by establishing different deep learning networks, including autoencoders (DAEs) [13], generative adversarial networks (GANs) [14], transfer learning (TL) [15] and so on. Those methods can achieve satisfactory denoising performance through continuous and extensive training and testing. However, this type of method requires a large amount of data to improve its denoising effectiveness, and the loss function based on cross entropy leads to a long training period, insufficient stability, and poor adaptability [16]. In addition, low-quality signals and insufficient samples limit the application and performance of abnormal sound detection for pressure pipeline systems.

To address the above issues and overcome these methods’ shortcomings, an improved entropy-based condition monitoring method is proposed for pressure pipelines through an improved noise reduction model and abnormal sound detection. The generative adversarial network (GAN) and denoising autoencoder (DAE) are used to create the improved noise reduction model, and the GAN is embedded in the DAE model to improve the information entropy of the loss function. This model is employed for noise reduction, and high-quality acoustic signals are obtained through adversarial training. In addition, the abnormal sound detection method is proposed based on the long short-term memory (LSTM) network, and the detection result is used for condition monitoring of the pressure pipeline. The noise reduction performance and accuracy of condition monitoring are evaluated through the pressure pipeline experimental platform. In addition, the superiority of the proposed method is tested by comparing it with the existing methods for noise reduction and condition monitoring. The main contributions include the following:

- The pressure pipeline condition monitoring method is proposed to detect abnormal sound based on an improved noise reduction model and abnormal sound detection.
- The improved noise reduction model is constructed by embedding the DAE and GAN with improved entropy, which can reduce noise and generate high-quality acoustic signals for different conditions.
- The pressure pipeline experimental platform is established to evaluate the performance and superiority of the proposed framework.

The remainder of this research is organized as follows. Section 2 introduces the theoretical background. The proposed improved entropy-based condition monitoring method is detailed in Section 3. Section 4 displays the pressure pipeline experimental platform and performance of the proposed method. The conclusion is presented in Section 5.

## 2. Theoretical Background

### 2.1. Denoising Autoencoder

**Figure 1:**
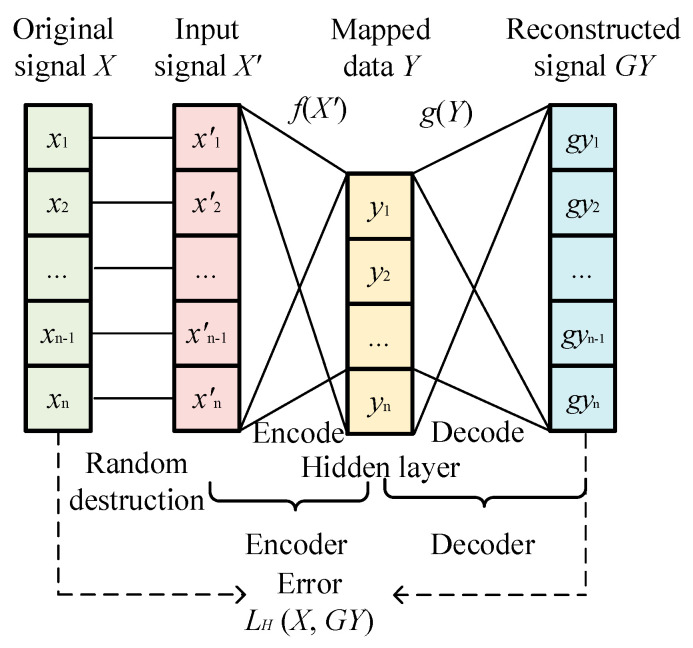
The structure and process of DAE.

As an unsupervised learning model, the auto-encoder directly encodes and decodes signals through an encoder and decoder. The encoder maps the original signal *X* to the hidden layer for the mapped data *Y*, which is decoded to obtain a reconstructed signal *GY* based on the decoder. By comparing the original signal *X* and the reconstructed signal *GY*, the error is calculated and the model is modified [17]. The loss function calculation during the process of encoding and decoding is expressed as

(1) $$\begin{array}{l} Y=f\left(X\right)=f\left(\omega_{1}X+b_{1}\right)\\ GY=g\left(Y\right)=g\left(\omega_{2}Y+b_{2}\right) \end{array},$$

where the *ω*_1_ and *b*_1_ are the weight coefficients and biases of the encoder, respectively; *ω*_2_ and *b*_2_ are the weight coefficients and biases of the decoder, respectively; and *X*, *Y*, and *GY* are the original signal, mapped data, and reconstructed signal, respectively.

(2) $$L_{H}(X,GY)=\frac{1}{n}\sum_{i=1}^{n}{\left(x_{i}-gy_{i}\right)}^{2},$$

where *L_H_* is the loss function, and *n* is the signal length. DAE is an improved version of the auto-encoder, which constructs the new input signal *X^′^* by randomly destroying the original signal *X*. Specially, the random noise is added to the original signal *X* to achieve random destruction, which is an input processing approach for the DAE to improve its stability and to overcome randomness. More detailed processes are introduced in [18]. It also employs the encoder and decoder for mapping and decoding, and the decoder is used to obtain reconstructed signal *GY*. In addition, the model is modified based on the difference between the input and output signal [19]. By utilizing the randomness of noise and the stability of real data, the robustness of DAE is improved through multiple random destruction and iterations, which is suitable for noise reduction. The structure and process of DAE is shown in Figure 1.

### 2.2. Generative Adversarial Network

**Figure 2:**
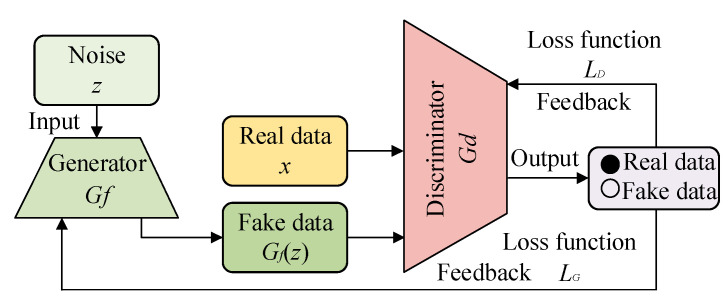
The structure of the GAN.

A GAN is an adversarial neural network model proposed by Goodfellow in 2014, including two networks: the generator and the discriminator [20]. The generator can generate deceptive fake data by inputting noise, while the discriminator is used to determine the difference between real and fake data to determine authenticity. Specifically, the output of the discriminator is the judgment for real data and fake data. Through continuous adversarial iterative training of the generator and discriminator, the performance of the generator continues to improve, and the quality of the fake data increases subsequently. However, the discriminative performance of the discriminator gradually weakens until it is unable to distinguish between real and fake data. At this point, adversarial iterative training ends [21]. Finally, the trained generator is employed to generate high-quality fake data. The structure of GAN is shown in Figure 2.

For the GAN, the adversarial process and loss function of the generator and discriminator can be expressed as

(3) $$\underset{G}{\min}\;\underset{D}{\max}V\left(G,D\right)=E_{x\sim p_{x}}\left[\log D\left(x\right)\right]+E_{z\sim p_{z}}\left[\log\left(1-D\left(G\left(z\right)\right)\right)\right],$$

where *x* and *G*(*z*) are the real and fake data, respectively; *E_x_*_~*Px*_ and *E_z_*_~*Pz*_ are the expectation of *x* and *G*(*z*), respectively; *x*~*P_x_* and *z*~*P_z_* are the generated distribution of *x* and *G*(*z*); respectively, and *G* and *D* are the generator and discriminator, respectively.

(4) $$\begin{array}{l} L_{G}=\log\left(1-D\left(G\left(z\right)\right)\right)\\ L_{D}=-\log\left(D\left(x\right)\right)+\log\left(1-D\left(G\left(z\right)\right)\right) \end{array},$$

where *L_G_* and *L_D_* are loss functions for the generator and discriminator, respectively.

## 3. Improved Entropy-Based Condition Monitoring for Pressure Pipeline

### 3.1. Improved Entropy-Based Noise Reduction Model

**Figure 3:**
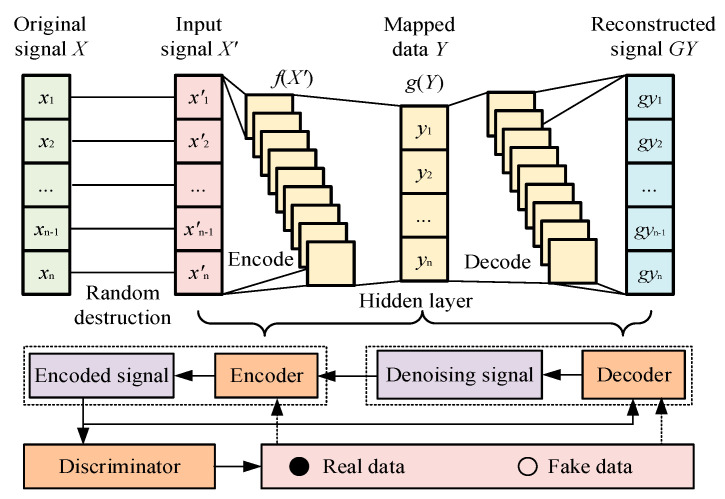
The structure of the proposed noise reduction model.

Based on the characteristics and disadvantages of the DAE, it is modified through the GAN, which constitutes the proposed improved entropy-based noise reduction model, as shown in Figure 3.

For this model, the generator of GAN is employed as the encoder of the DAE to generate the encoded signal, and the denoising signals are obtained from the decoder. For denoising process monitoring, the discriminator of GAN is employed by distinguishing the encoded and denoising signals. To improve the training efficiency and effectiveness, the loss function is modified with improved entropy. Through continuous adversarial training, the improved entropy-based noise reduction model is trained and modified, which is implemented to generate denoising signal through the trained decoder. The denoising process of the proposed improved entropy-based noise reduction model includes three parts, as follows: encode–decode–discriminator processing, training and feedback with improved entropy, and noise reduction.

**Figure 4:**
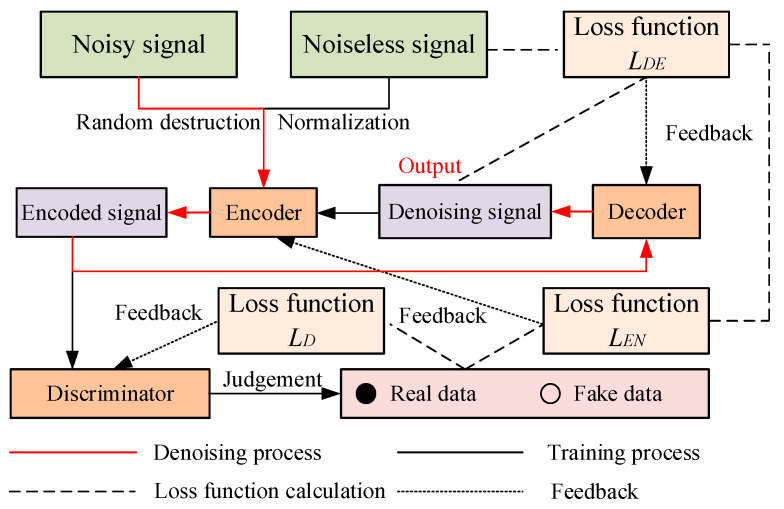
The process of training and noise reduction.

1. Encode–decode–discriminator processing: Based on the characteristic of the DAE, all original signals are randomly destroyed by adding random noise, and are normalized for the same amplitude range of [0,1]. Furthermore, the normalized signals are input into the encoder to generate encode signals, which are decoded by the decoder to obtain the denoising signals. To evaluate the denoising performance, the denoising signal obtained from the decoder is encoded again as the encoded signals by the encoder, which are analyzed by the discriminator. This process is employed to determine whether the data are real or fake.
2. Training and feedback with improved entropy: To evaluate the authenticity of the encoded signal and improve the quality of noise reduction, the loss function is calculated based on the judgment result of the discriminator. This process is employed to improve the entropy-based function, and conforms to the loss function of the GAN and generator, which is expressed as
  (5) $$L_{X^{\prime }}=\log\left(1-D\left(G\left(X^{\prime }\right)\right)\right),$$
  where *L_X’_* is the loss function for the input signal *X*’. For the proposed improved entropy-based noise reduction model, the loss function of the discriminator is improved through the result of signal judgment and is expressed as
  (6) $$L_{D}=-\log\left(D\left(X_{R}\right)\right)+\sum_{i=1}^{M}\log\left(1-D\left(G\left({X_{i}}^{\prime }\right)\right)\right),$$
  where *L_D_* is the loss function for the discriminator, *X_R_* is the real data (noiseless signal), *X_i_^′^* is the *i*-th input signal, and *M* is the number of input data types (including noisy and denoising signals). In addition, the root mean square error (RMSE) is employed to evaluate the effectiveness of the proposed improved entropy-based noise reduction model. The result is used as the loss function for the decoder, and is expressed as
  (7) $$L_{DE}=\sqrt{\frac{1}{n}\sum_{i=1}^{n}{\left(X_{Ri}-X_{Di}\right)}^{2}},$$
  where *L_DE_* is the loss function for the decoder; *X_Ri_* and *X_Di_* are the *i*-th values of the real data (noiseless signal) and denoising signal, respectively; and *n* is the signal length. Due to the generator being embedded in the DAE, the loss function of the encoder includes two aspects: DAE and GAN. This is calculated as follows:
  (8) $$L_{EN}=\sqrt{\frac{1}{n}\sum_{i=1}^{n}{\left(X_{Ri}-X_{Di}\right)}^{2}}+\sum_{i=1}^{M}\log\left(1-D\left(G\left({X_{i}}^{\prime }\right)\right)\right),$$
  where *L_EN_* is the loss function for the encoder. After calculating different loss functions with improved entropy, the results are fed back to the encoder, decoder, and discriminator. According to the process of adversarial training, the loss function of the proposed improved entropy-based noise reduction model is calculated and fed back during continuous iterations. Finally, adversarial training is completed when the encoder and discriminator reach adversarial balance, where the trained noise reduction model is obtained. The process of the adversarial training is expressed as
  (9) $$\underset{G}{\min}\;\underset{D}{\max}V\left(G,D\right)=E_{x\sim p_{x}}\left[\log D\left(X_{R}\right)\right]+E_{z\sim p_{z}}\left[\sum_{i=1}^{M}\log\left(1-D\left(G\left({X_{i}}^{\prime }\right)\right)\right)\right],$$
3. Noise reduction: Based on the trained improved entropy-based noise reduction model, the noisy acoustic signals are inputted into the trained encoder, and the denoising signals are obtained from the trained decoder, which are the output of the proposed model. The process of training and noise reduction are shown in Figure 4.

### 3.2. Abnormal Sound Detection

**Figure 5:**
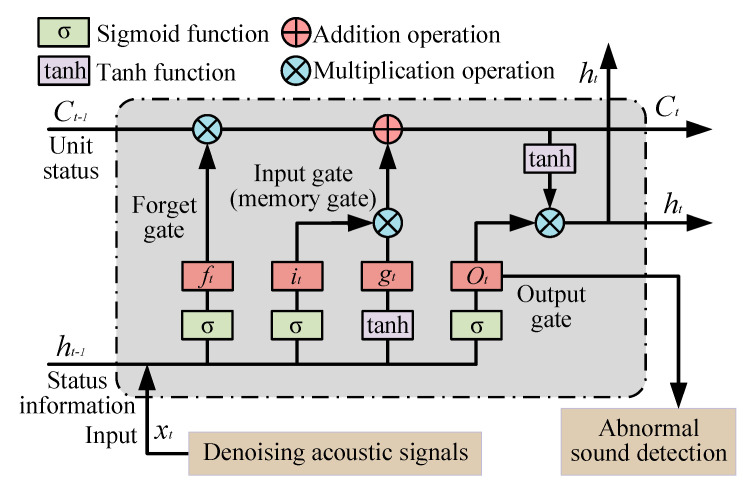
The structure of the real-time decision-making model.

Based on the improved entropy-based noise reduction model, high-quality acoustic signals are obtained as denoising monitoring signals. To realize condition monitoring for pressure pipelines, the abnormal sound detection method is proposed based on the LSTM. By inputting the denoising monitoring signal, the LSTM is trained for the forget gate and the input gate (memory gate). Specifically, the training of the LSTM is the same as in commonly used methods. Finally, the trained LSTM is employed to detect abnormal sound, and the results are applied for the condition monitoring of the pressure pipelines, as shown in Figure 5.

The denoising monitoring signals generated from the trained improved entropy-based noise reduction model are inputted into the forget gate with different labels and determine which information to discard based on the state information *h_t_*_−1_ at time *t* − 1 and the current input denoising acoustic signals *x_t_* at time *t*. This is expressed as

(10) $$f_{t}=\sigma \left(W_{f}[h_{t-1},x_{t}]+b_{f}\right),$$

where *W_f_* and *b_f_* are the weight and bias of forget gate, respectively; σ is the Sigmoid function; and *f_t_* is the output at time *t* for the forget gate. In addition, the input gate is used to process current information and update long-term states using the Tanh function. This is expressed as

(11) $$\begin{array}{l} i_{t}=\sigma \left(W_{i}[h_{t-1},x_{t}]+b_{i}\right)\\ g_{t}=\tanh\left(W_{g}[h_{t-1},x_{t}]+b_{g}\right) \end{array},$$

where *W_i_* and *b_i_* are the weight and bias of the Sigmoid function, respectively; *W_g_* and *b_g_* are the weight and bias of the Tanh function, respectively; and *i_t_* and *g_t_* are the output at time *t* for the Sigmoid and Tanh function, respectively. In addition, *i_t_* and *g_t_* are multiplied as the output information for the input gate. The current unit status *C_t_* is updated based on the historical unit status *C_t_*_−1_ and is expressed as

(12) $$C_{t}=f_{t}C_{t-1}+i_{t}g_{t},$$

After being processed by the Sigmoid function, the output *O_t_* is obtained for the condition monitoring of pressure pipeline, while the status information *h_t_* changes with the Tanh function at the output gate. This is expressed as

(13) $$\begin{array}{l} O_{t}=\sigma \left(W_{o}[h_{t-1},x_{t}]+b_{o}\right)\\ h_{t}=O_{t}\tanh\left(C_{t}\right) \end{array},$$

where *W_o_* and *b_o_* are the weight and bias of the output gate, respectively, and *O_t_* is the output at time *t* for the output gate.

### 3.3. Condition Monitoring for Pressure Pipeline

**Figure 6:**
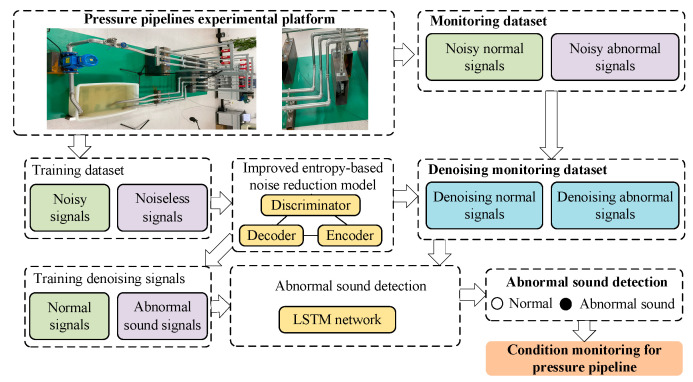
The process of the proposed pressure pipeline condition monitoring method.

Based on the improved entropy-based noise reduction model and result of abnormal sound detection, the condition monitoring method is proposed for a pressure pipeline, as shown in Figure 6. This includes the following steps:

1. Signal acquisition: Based on the pressure pipeline experimental platform, different types of acoustic signals are collected to establish the training and monitoring dataset. Specifically, the training dataset includes noisy and noiseless signals under one working condition, and the monitoring dataset consists of several noisy normal condition and abnormal condition signals under different working conditions.
2. Improved entropy-based noise reduction model training and application: The signals obtained from the training dataset are inputted into the improved entropy-based noise reduction model for training and testing. After adversarial learning, the trained model is applied for the training and monitoring dataset with noisy normal and abnormal signals, which can generate training denoising signals and a denoising monitoring dataset with denoising normal and abnormal signals.
3. Abnormal sound detection: Based on the training denoising signals obtained from the proposed improved entropy-based noise reduction model, the LSTM network is trained and tested. Finally, the trained LSTM network is employed for the denoising monitoring dataset for abnormal sound detection.
4. Condition monitoring for pressure pipeline: Based on the result of abnormal sound detection, the abnormal sound of the pressure pipeline is detected, which can realize condition monitoring for the pressure pipeline under different working conditions.

## 4. Experimental Platform and Condition Monitoring Results

### 4.1. Experimental Platform

**Figure 7:**
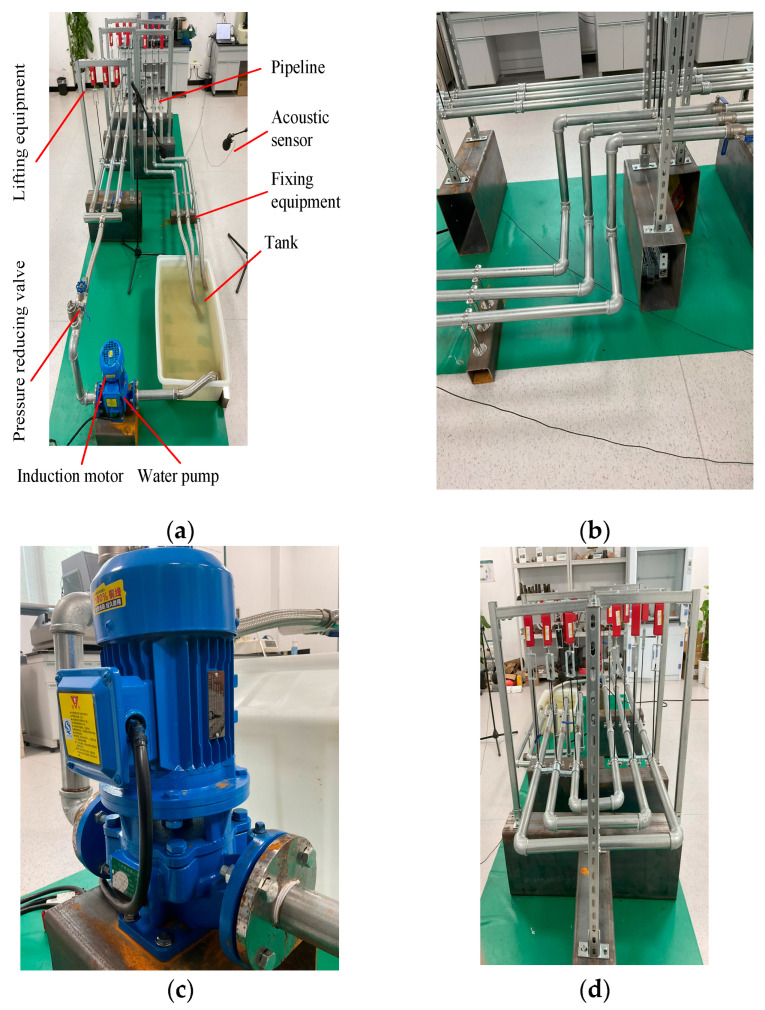
The equipment in the pressure pipeline experimental platform: (**a**) Experimental platform; (**b**) typical pipeline system; (**c**) induction motor, (**d**) lifting equipment.

To evaluate the effectiveness and reliability of the proposed method, a pressure pipeline experimental platform is established with several devices, as shown in Figure 7. This includes an induction motor, a water pump, a pressure reducing valve, pipelines, a tank, lifting and fixing equipment, and two acoustic sensors.

The water in the tank is pumped out by a water pump, and is regulated by a pressure reducing valve to move it along the pipeline. After passing through the experimental pipeline section, it flows back to the tank, which forms a closed loop. As shown in Figure 7b, the experimental pipeline section is a typical pipeline system, consisting of four pipelines with three degrees of freedom. The induction motor is a three-phase asynchronous motor of 0.75 Kw and 380 V with 2900 rpm, as shown in Figure 7c. To simulate the installation mode of the actual pipeline in a boiler system, the experimental pipelines are suspended in the air through the lifting equipment, which is fixed at the outlet of the pressure pipeline, as shown in Figure 7d.

Furthermore, the fixed device is loosened to simulate abnormal vibration of the pressure pipeline, thereby generating abnormal sound. Meanwhile, the acoustic sensor is employed to collect two types of signals in noisy and noiseless environments under three pressures: 5 PSI, 10 PSI and 15 PSI. The parameters of the experimental platform are shown in Table 1. To simulate the actual noise environment of pressure pipelines, the sound recorded at a boiler site is played during signal acquisition. The sampling frequency is 2560 Hz with 1 s, and each condition has 100 samples. Finally, there are 2 × 2 × 3 × 100 (2 conditions: normal and abnormal sound; 2 environments: noisy and noiseless; and 3 pressures: 5, 10 and 15 PSI) monitoring acoustic signals with 2560 data in each sample.

### 4.2. Results

**Figure 8:**
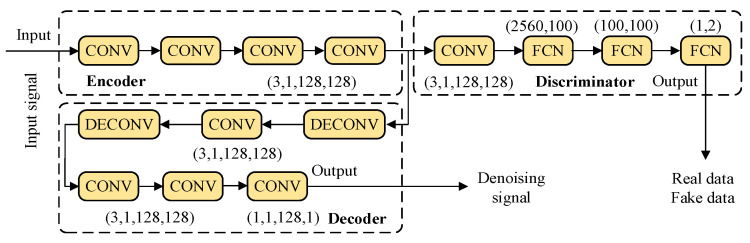
The structure and parameters of the proposed model.

Based on the length and characteristics of the acoustic samples, the parameters of the improved entropy-based noise reduction model are selected for the encoder, decoder and discriminator. The structure and parameter of the proposed model is shown in Figure 8. The DECONV, CONV, and FCN are the deconvolution, convolutional and fully connected layer, respectively.

The noisy and noiseless signal under one condition is employed to train the proposed improved entropy-based noise reduction model. According to the 7:3 ratio for training and testing, there are 70 and 30 training and testing samples under one condition. Specifically, the training epoch is 100, and the maximum batch is 32 with the adaptive gradient optimizer. After adversarial training, the improved entropy-based noise reduction model is trained and applied for noisy signals to generate denoising signals under the other two working conditions. To evaluate the denoising performance and verify the proposed model, the RMSE [22] and signal-to-noise ratio (SNR) [23] are employed as the evaluation feature to calculate the differences between noisy and denoising signals. This is expressed as

(14) $$RMSE=\sqrt{\frac{1}{n}\sum_{i=1}^{n}{\left(X_{Ri}-X_{Di}\right)}^{2}},$$

(15) $$SNR=10\mathrm{lg}\frac{{\sum_{i=1}^{n}{X_{D}}_{i}^{2}}_{S}}{\sum_{i=1}^{n}{\left(X_{Ri}-X_{Di}\right)}^{2}}$$

where *RMSE* is the value of the mean square error, and *SNR* is the value of the signal-to-noise ratio. Based on the RMSE and SNR, the performance of noise reduction is quantitatively analyzed. Taking normal samples as an example, the noise reduction effect of training and transfer under different working conditions is shown in Table 2. Clearly, the RMSE and SNR of noisy samples are higher and lower, denoting the existence of noise within the original samples, which has a significant effect on signal quality. After denoising using the proposed model, the RMSE is successfully reduced to less than 0.02, while the SNR can reach over 3.5 dB for samples at 15 PSI. This can increase by about 5 dB for the three conditions and further validates the efficacy and dependability of the proposed model.

Based on denoising acoustic signals, the abnormal sound detection method is applied for the condition monitoring of a pressure pipeline. Specifically, the LSTM network is employed with 200 hidden units, an epoch of 100, and a solver of the adaptive moment estimation with a gradient threshold of 1 and a dropping learning rate of 0.2. Furthermore, the parameters of the LSTM network are selected based on the actual experience and testing results [24]. By training and testing the LSTM through denoising signals, the abnormal sound is detected for the condition monitoring of pressure pipelines. By comparing the monitoring results, the noisy and noiseless samples are trained and tested directly with a ratio of 7:3. To avoid randomness, the result is the average value obtained by repeating the process five times, and is shown in Table 3 for three working conditions. Specifically, the mixture representing the trained denoising model is applied for all samples under three working conditions, which are trained and tested by the LSTM network to evaluate the proposed abnormal sound detection method.

Clearly, the accuracy of condition monitoring with noisy samples is only 68.33% for 10 PSI, which illustrates the negative impact of noise. Based on the proposed method, the accuracy is effectively improved for three conditions. Specifically, by training the improved entropy-based noise reduction model and LSTM network model with the signals collected at 5 PSI, the highest detection accuracy of 91.67% and 87.00% can be reached by applying the model to the signals collected at 5 and 10 PSI. The reason for this is that the noise is strong for low pressure, and that the anti-noise ability of the trained model is improved so as to be applicable to other conditions. When applying the trained model to high pressure with low noise, the accuracy is further improved for condition monitoring. Moreover, the precision of the proposed method is nearly equivalent to that of the noiseless samples and is about 15% higher than that of the noisy samples. This result further demonstrates the performance of the proposed condition monitoring method.

### 4.3. Discussions

**Figure 9:**
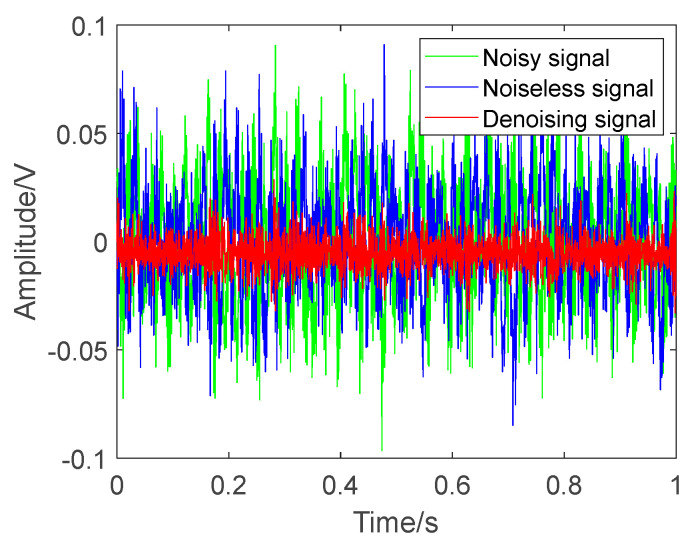
Noise reduction performance for three types of signals.

To test the performance of the proposed improved entropy-based noise reduction model, the original, denoising and noiseless acoustic signals are compared based on the proposed and original method in relation to time frequency. Taking the acoustic sample at 5 PSI as an example, the three types of signals are shown in Figure 9. The noisy signal has a higher value that is more disordered. After processing with the proposed improved entropy-based noise reduction model, the amplitude of the acoustic signal decreases via the reduction of noise, and the result is closer to that for the noiseless signals.

**Figure 10:**
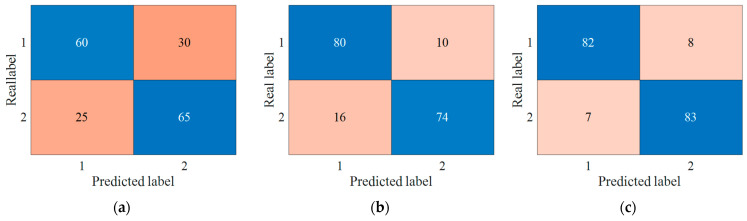
Confusion matrix for three types of signals. (**a**) Noisy samples; (**b**) denoising samples; (**c**) noiseless samples.

To intuitively reflect the accuracy and bias of the abnormal sound detection method for condition monitoring, and while taking the one result of the mixture condition with 5 PSI training as an example, the confusion matrix for the three types of signals is shown in Figure 10. As the y-axis represents the number of real samples, the sum of the elements in each row in the confusion matrix is equal for each class with 90 samples. Due to the interference of noise, the accuracy of noisy samples is the lowest, and there are also more cases of false detection. Based on the denoising samples obtained from the proposed improved entropy-based noise reduction model, the accuracy is improved, and some false conditions are modified. The result of the proposed abnormal sound detection is close to that of noiseless samples and illustrates the effectiveness of the proposed method. Furthermore, the false samples are basically equal for normal and abnormal sound signals, which proves the stability and balance of the proposed method.

In addition, the comparative analysis is implemented through different methods, including the signal processing method and denoising model. Specifically, the EMD [9] and WPD [11] are employed as the signal processing methods, while the DAE [13] and CGAN [25] are used as the noise reduction models. The RMSE and SNR are employed as the features by which to measure the performance in noise reduction, and the results are shown in Table 4. Specifically, the results are the average values obtained by applying the model to different pressures.

Clearly, the original noisy samples have poor RMSE and SNR due to their complex noise. By processing with the EMD and WPD, the signal quality is improved, and the RMSE can decrease about 0.11 for the three conditions. Specifically, the result of EMD is little better than the WPD. Based on different denoising models, the results of DAE and CGAN display effective performance in noise reduction, providing better results than the WPD. Furthermore, the results of the CGAN model are unstable due to the complexity and uncertainty of the input noise. Compared with other methods, the denoising signals generated by the proposed method reach a smaller RMSE and higher SNR, and the result is relatively stable in various conditions. This result quantitatively demonstrates the performance of the proposed method.

To analyze its effectiveness, the accuracy of condition monitoring is calculated for different approaches, and the result is shown in Table 5. Specifically, for noisy and noiseless samples, the accuracy of one condition is informed by the detection results obtained by directly inputting signals into the LSTM network for training and testing under the same condition. Furthermore, the EMD and WPD form the same application process.

The accuracy of abnormal sound detection is the lowest for the noisy sample, and has been improved by reducing the noise of noisy signals through different methods. The denoising methods based on EMD and WPD can improve the accuracy by about 10% for some conditions. For the denoising model-based method, about 80% detection accuracy is obtained, similar to the DAE and the CGAN. This indicates the effectiveness of the denoising model. In addition, the proposed method can achieve an accuracy of about 85% for different conditions, which approximates the outcome of noiseless samples and surpasses other methods. This can improve the accuracy of abnormal sound detection and condition monitoring by reducing noise in the acoustic signal, further verifying the effectiveness of the proposed method.

## 5. Conclusions

This paper proposes a condition monitoring method for pressure pipeline based on an improved entropy-based noise reduction model and an abnormal sound detection method. It establishes an improved entropy-based noise reduction model based on the DAE and GAN, which is trained by comparing the noisy and noiseless acoustic signals under single working condition. By means of continuous adversarial training with the modified loss function of improved entropy, the denoising ability of the noise reduction model is improved, which is applied to different conditions in order to obtain high-quality denoising signals. Further, an abnormal sound detection method is proposed based on the LSTM network, one which realizes the condition monitoring of pressure pipelines through a complete denoising signals dataset. Based on the experimental platform, the effectiveness and reliability of the proposed method is tested and evaluated for three pressure conditions. The results indicate that the proposed method can effectively reduce the noise of the noisy signals and reach about 92% condition monitoring accuracy for different conditions, which is approximately equal to the accuracy of noiseless samples. Furthermore, the ablation experiments and comparative analysis are conducted to verify the superiority of the proposed method, and the result further demonstrates its effectiveness and performance.

In the future, more abnormal sound conditions can be simulated and tested to generate abnormal acoustic signals, which is the extension for implementing condition monitoring. In addition, there is a certain gap in the noise between experimental and actual signals. By monitoring the abnormal sound obtained from the actual conditions, the applicability and stability of the proposed method can be further tested.

## Figures and Tables

**Table 1:** The parameter of the experimental platform.

Type	Value
Pressure/PSI	5, 10 and 15
Sampling frequency/Hz	2560
Environments	Noisy and noiseless
Conditions	Normal and abnormal sound

**Table 2:** Noise reduction effect of training and transfer under different working conditions.

Evaluation Index	Sample Type	Training at 5 PSI		Training at 10 PSI		Training at 15 PSI	
		10 PSI	15 PSI	5 PSI	15 PSI	5 PSI	10 PSI
RMSE	Noisy	0.039	0.037	0.058	0.037	0.058	0.039
	Denoising	0.017	0.008	0.019	0.007	0.020	0.016
SNR	Noisy	−2.55	−2.18	−3.02	−2.18	−3.02	−2.55
	Denoising	3.21	3.56	2.58	3.47	2.51	3.15

**Table 3:** The condition monitoring results for pressure pipeline.

Conditions		Denoising Samples	Noisy Samples	Noiseless Samples
Denoising Training	Application			
5 PSI	5	91.67%	71.67%	93.33%
	10	86.33%	68.33%	90.67%
	15	87.00%	73.33%	90.33%
	Mixture	85.67%	69.33%	91.67%
10 PSI	5	84.67%	71.67%	93.33%
	10	89.33%	68.33%	90.67%
	15	86.33%	73.33%	90.33%
	Mixture	84.67%	69.33%	91.67%
15 PSI	5	85.33%	71.67%	93.33%
	10	84.33%	68.33%	90.67%
	15	88.67%	73.33%	90.33%
	Mixture	85.67%	69.33%	91.67%

**Table 4:** The comparative denoising results for different methods.

Methods	Training at 5 PSI		Training at 10 PSI		Training at 15 PSI	
	RMSE	SNR	RMSE	SNR	RMSE	SNR
Noisy samples	0.207	−1.96 dB	0.221	−2.60 dB	0.230	−2.38 dB
EMD	0.173	1.43 dB	0.187	−0.86 dB	0.196	−1.09 dB
WPD	0.184	−0.73 dB	0.195	−1.15 dB	0.200	−1.21 dB
DAE	0.152	1.67 dB	0.155	1.93 dB	0.161	1.79 dB
CGAN	0.167	1.45 dB	0.145	1.95 dB	0.176	1.83 dB
The proposed method	0.114	3.39 dB	0.114	3.03 dB	0.134	2.83 dB

**Table 5:** The comparative condition monitoring results for different methods.

Methods	Training at 5 PSI		Training at 10 PSI		Training at 15 PSI	
	10 PSI	15 PSI	5 PSI	15 PSI	5 PSI	10 PSI
Noisy samples	68.33%	73.33%	71.67%	73.33%	71.67%	68.33%
Noiseless samples	90.67%	90.33%	93.33%	90.33%	93.33%	90.67%
EMD	84.67%	83.67%	82.67%	83.67%	82.67%	84.67%
WPD	84.33%	82.67%	83.33%	82.67%	83.33%	84.33%
DAE	78.33%	82.00%	78.00%	80.33%	78.00%	81.33%
CGAN	82.67%	80.67%	80.33%	82.33%	81.67%	70.33%
The proposed method	86.33%	87.00%	84.67%	86.33%	85.33%	84.33%

## Data Availability

The data that support the findings of this study are available on request from the corresponding author.
